# IL-36 signalling enhances a pro-tumorigenic phenotype in colon cancer cells with cancer cell growth restricted by administration of the IL-36R antagonist

**DOI:** 10.1038/s41388-022-02281-2

**Published:** 2022-04-01

**Authors:** Kevin Baker, Charlotte O’Donnell, Maura Bendix, Samuel Keogh, James Byrne, Michael O’Riordain, Peter Neary, Aileen Houston, Elizabeth Brint

**Affiliations:** 1 Departments of Pathology, University College Cork, Cork, Ireland; 2 APC Microbiome Ireland, University College Cork, Cork, Ireland; 3 Departments of Medicine, University College Cork, Cork, Ireland; 4 Cancer Research@UCC, University College Cork, Cork, Ireland; 5 Mercy University Hospital, Cork, Ireland; 6 Waterford University Hospital, Waterford, Ireland

**Keywords:** Cancer microenvironment, Cytokines

## Abstract

The IL-36 cytokines are a recently described subset of the IL-1 family of cytokines, shown to play a role in the pathogenesis of intestinal diseases such as Inflammatory Bowel Disease (IBD). Given the link between IBD and colitis –associated cancer, as well as the involvement of other IL-1 family members in intestinal tumorigenesis, the aim of this work was to investigate whether IL-36 cytokines play a role in the pathogenesis of colon cancer. Whilst research to date has focused on the role of IL-36 family members in augmenting the immune response to induce tumour rejection, very little remains known about IL-36R signalling in tumour cells in this context. In this study we demonstrate that expression of IL-36 family member mRNA and protein are significantly increased in colorectal cancer tissue compared to adjacent non-tumour. In vitro assays showed stimulation of colon cancer cell lines with IL-36R agonists resulted in the activation of the pro-tumorigenic phenotypes of increased cellular migration, invasion and proliferation in both 2D and 3D models. In addition, the IL-36 cytokines induced strong expression of pro-inflammatory chemokines in both human and murine cell lines. Intraperitoneal injection of IL-36Ra significantly reduced tumour burden using the subcutaneous CT26 tumour model in syngeneic Balb/mice, and this was associated with a decrease in Ki-67 expression by tumour cells in the IL-36Ra- treated group relative to untreated, suggesting the inhibition of the pro-proliferative signalling of IL-36 agonists resulted in the decreased tumour size. Moreover, colon cancer cells lacking the IL-36R also showed reduced tumour growth and reduced Ki-67 expression in vivo. Taken together, this data suggests that targeting IL-36R signalling may be a useful targeted therapy for colorectal cancer patients with IL-36R^+^ tumour cells.

## Introduction

The IL-36 cytokines are a subset of the IL-1 family of cytokines [1, 2]. The three agonistic members of this family, IL-36α, IL-36β and IL-36γ, all share the same receptor complex, which is composed of the IL-36 receptor (IL-36R/IL1RRP2/IL1RL1) and the IL1 Receptor accessory protein. A biological inhibitor to this complex has also been identified, the IL-36R antagonist (IL-36Ra). The IL-36 cytokines and their receptor are expressed by several tissues, particularly the lung, skin and intestine, as well as by immune cells such as monocytes, macrophages, dendritic cells (DCs) and T cells [3, 4]. Similar to other IL-1 family members, IL-36 cytokines are important activators of the inflammatory response, stimulating both innate and adaptive immune responses [3, 4]. These cytokines have been shown to play an important role in autoimmune diseases, in particular in the pathogenesis of psoriasis [3, 5], Inflammatory Bowel Diseases [6, 7] and respiratory diseases [8]. Given that inflammation is now recognised as a hallmark of cancer, IL-36 is now being increasingly investigated in, and implicated in, multiple cancer types.

Initial studies investigated the role of IL-36γ in melanoma and metastatic breast cancer. B16 melanoma cells engineered to overexpress IL-36γ displayed reduced tumour growth and improved prognosis, with IL-36γ promoting the activation and proliferation of CD8^+^ cells and NK cells [9] compared to B16 control-injected mice. Using a similar model involving the 4T1 breast cancer cell line, IL-36γ overexpression promoted Th_1_ anti-tumorigenic responses, reduced tumour size and reduced the number of metastatic pulmonary lesions. The IL-36γ-mediated anti-tumorigenic response was characterised by a reduction in tumour-promoting B cells, a reduction in Gr1^+^ neutrophilic myeloid-derived suppressor cells (MDSCs) and enhanced expression of MHC class II molecules across all MDSC subsets. More recently, the anti-tumorigenic effects of IL-36β were also examined in B16 and 4T1 cells in vivo and similar findings were observed, although in this study the ability of IL-36β to promote the activation of CD8^+^ T cells was shown to be dependent on mTORC1 activation [10]. In addition, IL-36 signalling in cancer has been shown to promote the development and sustenance of Tertiary Lymphoid Structures (TLO) in the tumour microenvironment (TME) [11]. TLOs trigger anti-tumorigenic responses by promoting DC-mediated tumour antigen presentation and T-cell priming. Elevated IL-36γ expression was associated with TLO formation, enhanced CD20^+^ B cells and CD4^+^ memory T-cell infiltration and overall better patient prognosis [11]. The injection of tumours with DCs engineered to secrete a bioactive form of mIL-36γ also initiated therapeutic TLO and slowed tumour progression in vivo[12]. In addition to the generation of TLOs, the ability of IL-36 cytokines to promote an anti-tumorigenic immune response has been further reinforced by recent findings showing that IL-36β can enhance CD8^+^ T-cell proliferation and activation [10, 13]. Overall, therefore, the mechanism of action of IL-36 in driving tumour suppressive effects appears to be via modification of the TME and the promotion of an anti-tumorigenic immune response. In contrast, the direct role of IL-36 signalling in tumour cells is relatively underexplored.

It is well established that Ulcerative Colitis (UC) can predispose to the development of colon cancer (CRC) [14]. Recent findings have indicated a key role for IL-36 in the pathogenesis of UC. Expression of IL-36α and IL-36γ levels are elevated in UC patients whereas, IL-36Ra levels are attenuated [6, 15–18]. IL-36α and IL-36γ drive epithelial inflammatory responses and contribute to epithelium proliferation and upregulation of matrix remodelling protease expression (17). Despite these findings, little is known regarding the role of IL-36 in colorectal (CRC). Whilst few studies have directly examined the role of IL-36 in CRC, low levels of IL-36γ was shown to correlate with better CRC patient prognosis. Of note, however, in the same study, elevated expression of IL-36α was shown to be associated with better patient prognosis and increased CD3^+^ and CD8^+^ T-cell infiltration [19]. In contrast to the anti-tumorigenic role for IL-36 shown in breast cancer and melanoma (9, 10), most recently, using a series of murine models, IL-36γ was shown to promote intestinal inflammation and colon cancer development [20]. Specifically, IL-36γ upregulated extra-cellular matrix adhesion molecules and facilitated Wnt signalling. Together these findings highlight the complexity of the IL-36 family in intestinal disease.

Here we show that human CRC specimens express the IL-36R and that stimulation of colon cancer cell lines drives production of pro-tumorigenic chemokines, cellular proliferation, migration and invasion. Administration of IL-36Ra reduced tumour growth in vivo, as did ablation of expression of the IL-36R on tumour cells. Collectively this data indicates that IL-36 signalling is capable of inducing a pro-tumorigenic phenotype in IL-36R-expressing colon cancer cells, and thus should be considered carefully given the emerging data demonstrating an anti-tumorigenic function for IL-36R agonists within the TME through augmentation of the anti-tumour immune response.

## Results

### Expression of IL-36 family members is increased in colon cancer

**Fig. 1:**
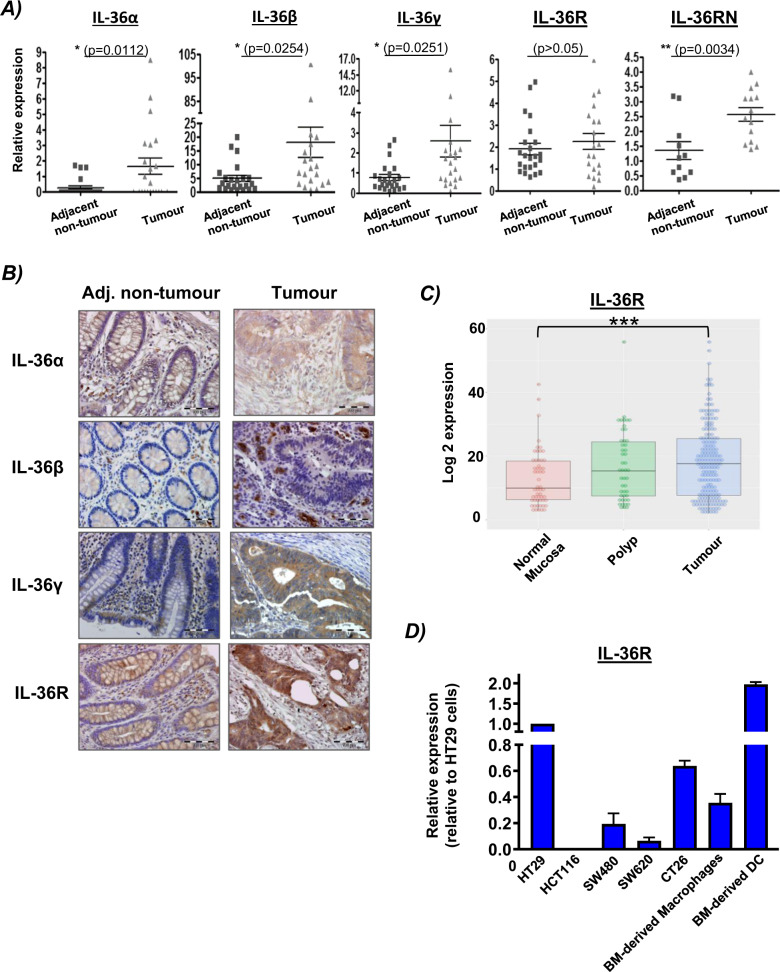
Expression of IL-36 family members is increased in colon cancer. **A** RNA was extracted from human colon cancer tissue and adjacent non-tumour tissue (*n* = 24), and gene expression of IL-36α, IL-36β, IL-36γ, IL-36RN and IL-36R were determined by qRT-PCR. **B** Paraffin-embedded human colon cancer tissue and adjacent non-tumour tissue (*n* = 66) were immunostained using anti-IL-36α, anti-IL-36β, anti-IL-36γ or anti-IL36R. Representative images are shown (mag = 40X; Scale 100 µm). **C** Affymetrix microarray data from the Gene Expression Omnibus (GSE68468) was analysed for IL-36R expression in normal colon mucosa, polyp tissue and colon cancer tissue. **D** RNA was extracted from colon cancer cell lines and murine bone marrow (BM)-derived macrophages and dendritic cells (DC), normalised, and expression of IL-36R analysed by RT-PCR. Statistical analysis was performed by Student T Test (**A**) and One-way ANOVA (**C**). *p* < 0.05, **p < 0.01, ***p < 0.001.

Expression of IL-36 family members in CRC was initially investigated. Using a cohort of 24 patient samples (patient cohort 1), gene expression was first examined by qRT-PCR. Expression of all family members, with the exception of the IL-36R, was found to be significantly upregulated in tumour tissue relative to adjacent non-tumour tissue (Fig. 1A). Protein expression of these family members was analysed by IHC (patient cohort 2) in both tumour and adjacent non tumour tissue. IL-36α and IL-36γ were detected at a very low level in the adjacent normal epithelial cells and stromal cells, with a higher level of expression detected in the tumour cells. IL-36β was only detected in the stromal compartment. The IL-36R was detectable in both the epithelial and stromal compartments in both adjacent normal and tumour tissue. To provide a semi-quantitative analysis of the changes in IL-36 family member expression, tumour cell and adjacent normal epithelial cell expression of IL-36α, IL-36γ and the IL-36R was scored based on intensity of staining. Findings were reflective of the qRT-PCR data, showing an increase in expression of these IL-36 family members relative to adjacent normal (Table 1). Changes in expression did not correlate with increasing tumour stage. Mining of the NCBI Microarray dataset GSE68468 also revealed that IL-36R expression increased with tumour development, being upregulated in polyp and tumour tissue in comparison to normal colonic mucosal tissue (Fig. 1C). Finally, RNA was extracted from multiple colon cancer cell lines and expression of the IL-36R assessed by qRT-PCR. IL-36R gene expression was shown to be highly expressed in HT29 and CT26 cells, which were, therefore, selected as the cell lines of choice for subsequent experimentation (Fig. 1D).

**Table 1:** IL-36 family member Expression in Colon Tumour cells and Adjacent normal epithelial cells.

		0-negative	1-weak	2-moderate	3-strong
IL-36α	Adj. Normal	48 (73%)	16 (24%)	2 (3%)	0
	Tumour	28 (42%)	18 (27%)	14 (21%)	6 (10%)
IL-36γ	Adj. Normal	40 (61%)	26 (39%)	0	0
	Tumour	2 (3%)	18 (27%)	26 (39%)	20 (31%)
IL-36R	Adj. Normal	24 (36%)	42 (64%)	0	0
	Tumour	3 (5%)	33 (50%)	20 (30%)	10 (15%)

Tumour cells and adjacent normal epithelial cells were scored as negative, weak, moderate or strong, according to the intensity of IL-36α, IL-36γ and IL-36R expression. (*n* = 66). % represents the number of sections scored in the corresponding category as a percent of the total number of samples scored.

### IL-36R agonists increase the expression of cytokines/chemokines by colon cancer cells

**Fig. 2:**
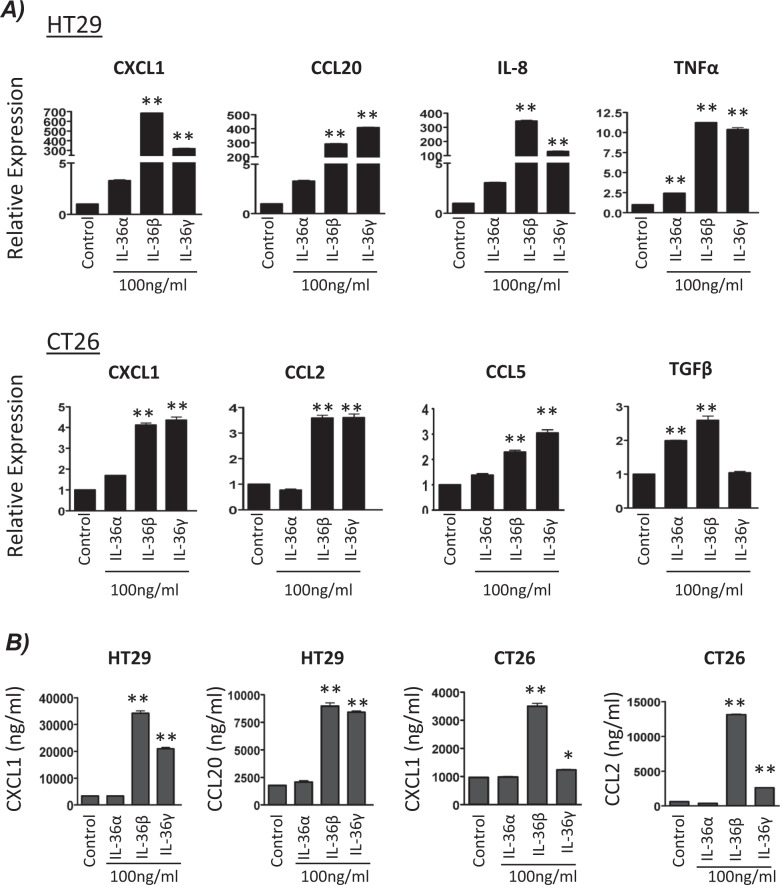
IL-36R agonists increase the expression of cytokines/chemokines by colon cancer cells. **A** Cells were stimulated with 100 ng/ml of IL-36α, IL-36β or IL-36γ. Changes in cytokine/chemokine expression were detected by qRT–PCR after 4 h and (**B**) by ELISA after 24 h. Data shown are mean ± s.e.m. (*n* = 3 independent experiments) and analysed by One-way ANOVA with Dunnetts post hoc test. *p* < 0.05, **p < 0.01.

In order to characterise the role of IL-36R signalling in CRC, cell lines were stimulated with IL-36R agonists and inflammatory gene expression change quantified by qRT-PCR and ELISA. In the human-derived HT29 cell line, IL-36β and IL-36γ were shown to be more potent inducers of inflammatory genes in comparison to IL-36α stimulation, with all genes showing greater induction in response to IL-36β and IL-36γ. Similar findings were seen in the mouse-derived cell line, CT26 (Fig. 2A). The ability of the IL-36 agonists to augment inflammatory responses in these cell lines were confirmed by detection of CXCL1, CCL2 and CCL20 protein. The ELISA data was reflective of the mRNA expression findings, once again showing IL-36β and IL-36γ to be much more potent in driving gene expression and subsequent protein synthesis (Fig. 2B).

### IL-36R agonists induce the proliferation of colon cancer cells

**Fig. 3:**
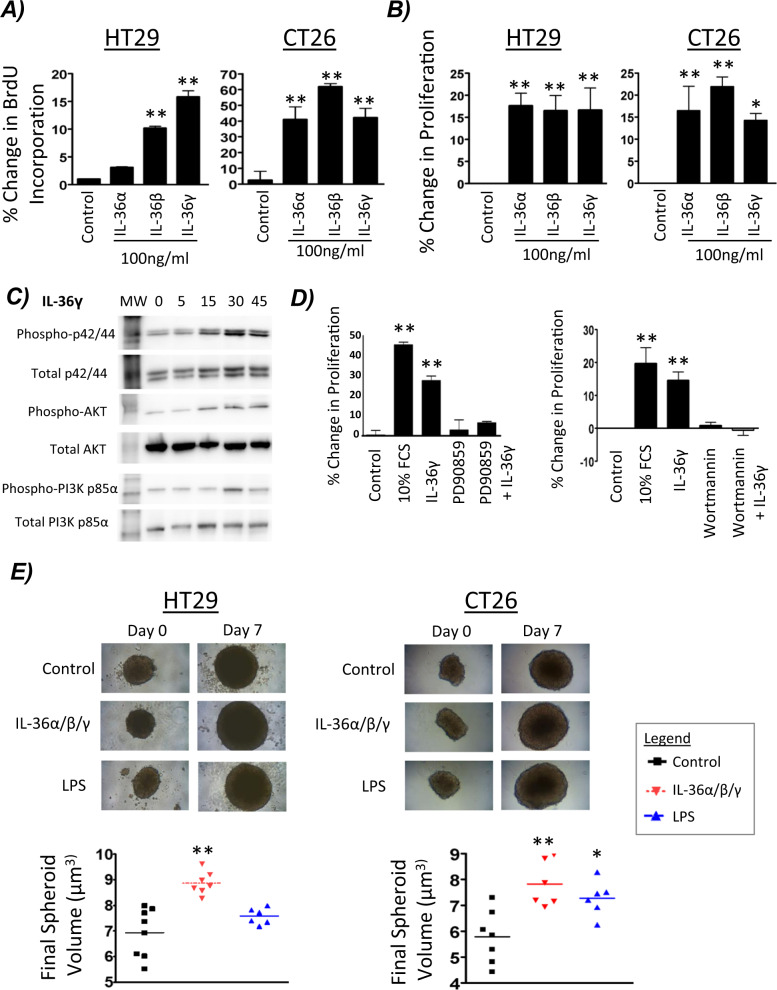
IL-36R agonists induce the proliferation of colon cancer cells. **A** Cells were stimulated for 24 h with 100 ng/ml of IL-36α, IL-36β or IL-36γ and changes in proliferation detected by BrdU incorporation or (**B**) by Resazurin reduction. **C** Cells were stimulated with IL-36γ as a time course over 60 min, subsequently lysed and examined for protein phosphorylation by Western Blot. **D** Cells were stimulated with IL-36γ for 24 h with/without PD90859 (10 μM) or Wortmannin (0.5 μM) pre-treatment and cellular proliferation was measured by resazurin reduction. **E** Spheroid formation was investigated in agar-coated flat bottom 96 well plates. Cells were stimulated with 100 ng/mL of a cocktail of IL-36α/b/y or with 100 ng/mL of LPS as a positive control. Graphs are representative of 3 biological replicates, consisting of 6 technical replicates per group. Data shown are mean ± s.e.m. (*n* = 3) and analysed by One-way ANOVA with Dunnetts post hoc test. *p* < 0.05, **p < 0.01.

In order to further investigate the role of IL-36R signalling on colon cancer cells, cells were stimulated with the three IL-36R agonists and studied for changes in cellular proliferation in both 2D and 3D models. Changes in BrdU incorporation and resazurin reduction were measured in both HT29 and CT26 cell lines. Each of the IL-36R agonists significantly increased cellular proliferation as assessed by both BrdU incorporation (Fig. 3A) and resazurin reduction (Fig. 3B). In order to examine through which signalling pathway IL-36 may be mediating cellular proliferation, Western blotting was performed on HT29 cells stimulated with IL-36γ, given it was the family member that consistently drove cellular proliferation to the greatest extent in HT29 cells. The p42/44 MAPK and pI3-kinase/AKT pathways were selected for investigation as they are both strongly associated with cellular proliferation [21] and have previously been shown to be activated by the IL-36 receptor signalling in other cell types [10]. The p42/44 MAPK pathway and the PI3K/AKT pathways were shown to be activated in response to IL-36γ stimulation (Fig. 3C), with IL-36R agonist-induced cellular proliferation shown to be inhibited upon incubation of cells with the inhibitors of these pathways, PD98059 and Wortmannin, respectively (Fig. 3D).

In order to more closely recreate primary tumours in vitro, single-mass spheroids were generated and measured for changes in size in response to continued stimulation with an IL-36R agonist cocktail containing equal amounts of all three cytokines. Both HT29 and CT26 spheroids significantly increased in size following IL-36 agonist stimulation when compared to the untreated control and the positive pro-inflammatory control LPS (Fig. 3E).

### IL-36R agonists increase colon cancer cell migration and invasion

**Fig. 4:**
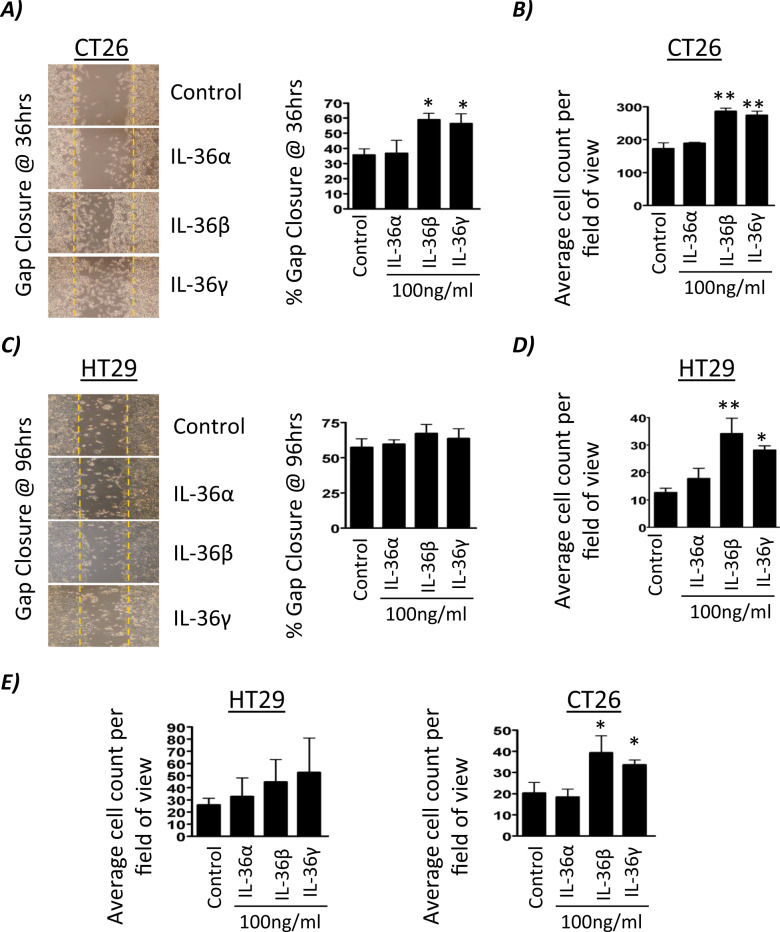
IL-36R agonists increase colon cancer cell migration and invasion. CT26 or HT29 cells were stimulated with IL-36α, IL-36β or IL-36γ (100 ng/mL) and changes in migration assessed using the Wound Scratch assay (**A**, **C**) and modified Boyden chamber assay (**B**, **D**). Cellular invasion was assessed using a Matrigel invasion assay system (**E**) and findings displayed as average cell count per field of view. Images are representative of three independent experiments. Data shown are mean ± s.e.m. (*n* = 3) and analysed by One-way ANOVA with Dunnetts post hoc test. *p < 0.05, **p < 0.01.

As the ability of IL-36 agonists to drive colon cancer cell proliferation indicated a possible pro-tumorigenic role effect of these cytokines on cancer cells, we next sought to investigate whether IL-36 signalling could alter cellular migration and invasion in vitro. Wound scratch migration assays showed IL-36β and IL-36γ were able to rapidly induce cellular migration in CT26 cells compared to the untreated control (Fig. 4A). Boyden-chamber Transwell migration assays were used to confirm wound scratch findings, with similar patterns of cellular migration observed (Fig. 4B). IL-36β and IL-36γ stimulation marginally increased wound scratch closure in HT29 cells, although at a much slower rate than CT26 cells reflecting a slower rate of growth (Fig. 4C). However, increased migration of HT29 cells in response to IL-36β and IL-36γ stimulation was observed to be significant using the Boyden-chamber Transwell assay (Fig. 4D). Cellular invasion assays were performed using Matrigel coated Boyden chambers. Whilst HT29 cells showed a trend of increased invasion following IL-36R agonist stimulation, IL-36β and IL-36γ showed a significant increase in cellular invasion in CT26 cells when compared to the untreated control and also IL-36α (Fig. 4E).

### Administration of IL-36RN and IL-36 agonist cocktail reduces tumour burden

**Fig. 5:**
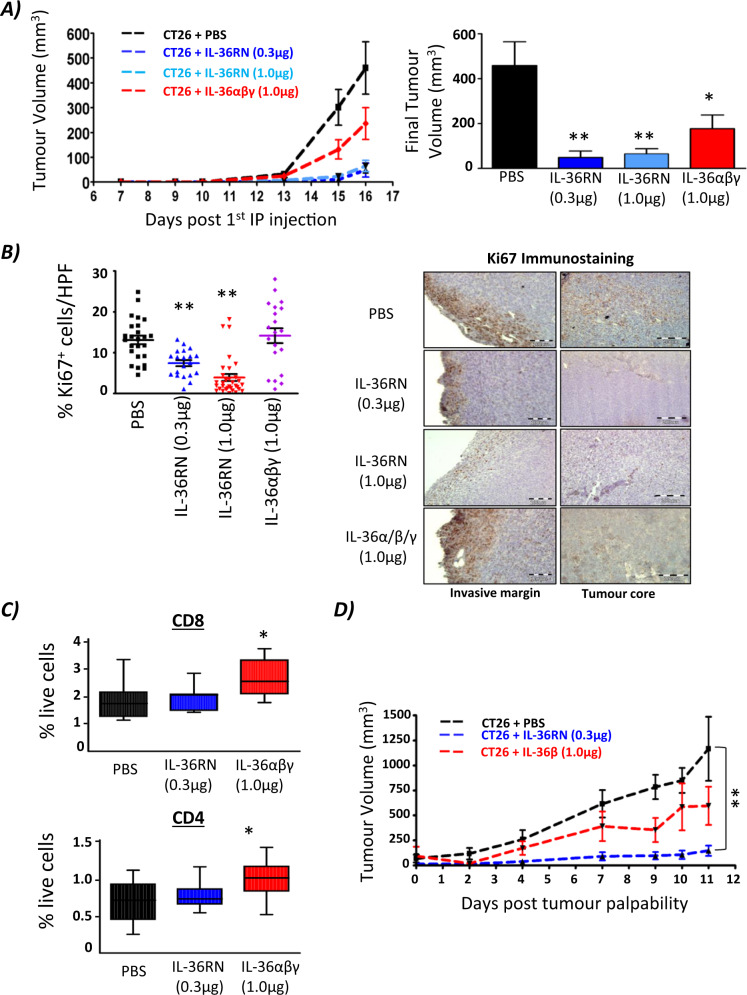
Administration of IL-36RN suppresses tumour growth in vivo. **A** CT26 cells (2 × 10^5^) were subcutaneously inoculated into BALB/c mice (*n* = 7 per group, day 0). 7 days later, IL-36Ra (0.3 µg or 1.0 µg), IL-36α/β/γ (1/0 µg each) or PBS was administered by IP injection twice weekly, and tumour growth monitored by regular measurement of tumour length (a) and width (b) using a Vernier calliper, and the volume was calculated as follows: ½ (a x b^2^). Data points represent the mean value ± SEM with final tumour volumes also shown. **B** Tumour tissue was extracted, processed, paraffin-embedded and subsequently immunostained with anti-Ki67. Representative images are shown (mag = 40X; Scale 100 µm). Images were taken of invasive margins and tumour cores and quantified by ImageJ software. **C** Flow cytometric analysis was performed on tumour tissue on the day of tumour extraction for immune cell infiltration quantification. **D** CT26 cells (2 × 10^5^) were subcutaneously inoculated into BALB/c mice. Following the detection of palpable tumours, IL-36Ra (0.3 µg), IL-36β (1.0 ug) or PBS was administered by IP injection twice weekly. Tumour growth was monitored as previously described. Data points represent the mean value ± SEM. **p < 0.01 and analysed by One-way ANOVA with Dunnetts post hoc test.

We next investigated the biological effect of inhibiting and augmenting systemic IL-36R signalling in vivo in immunocompetent Balb/c mice. Subcutaneous injection of CT26 cells in the rear flank of mice was followed by IP injection of PBS (control), IL-36Ra (0.3 ug or 1.0 ug) or an IL-36R agonist cocktail (1 ug in total) twice weekly. Tumour volume was observed to be significantly decreased in mice treated with IP injections of both IL-36Ra and IL-36R agonist cocktail when compared to the PBS control group. However, administration of the IL-36Ra resulted in a far greater reduction in tumour volume compared to the IL-36R agonist cocktail (Fig. 5A). In line with our findings that IL-36 agonists drive proliferation of tumour cells, IHC for Ki-67 as a marker of cellular proliferation revealed significantly decreased Ki-67 positive cells in both IL-36Ra-treated groups in comparison to both the control group and IL-36R agonist cocktail-treated groups, with representative images shown in Fig. 5B. Flow cytometric analysis showed no observable change in immune populations in IL-36Ra-treated groups in comparison to the PBS control. However, the IL-36R agonist-treated group showed an increase in infiltrating CD8^+^ and CD4^+^ T cells in comparison to all groups, indicating that whilst both treatment regimens reduce tumour volume in vivo, alterations in tumour infiltrating immune populations were only observed in the IL-36R agonist-treated group (Fig. 5C). In contrast, no alterations in infiltration by macrophages and neutrophils were detected. In order to investigate the translational potential of these findings, mice were injected subcutaneously with CT26 cells followed by IP injection of IL36RA or IL-36β once the majority of mice had palpable tumours. Once again, both treatments resulted in a reduced tumour burden with IL-36RN administration again shown to be the more effective treatment of the two (Fig. 5D).

### Knockout of IL-36R in CT26 cells results in a reduced tumour burden in vivo

**Fig. 6:**
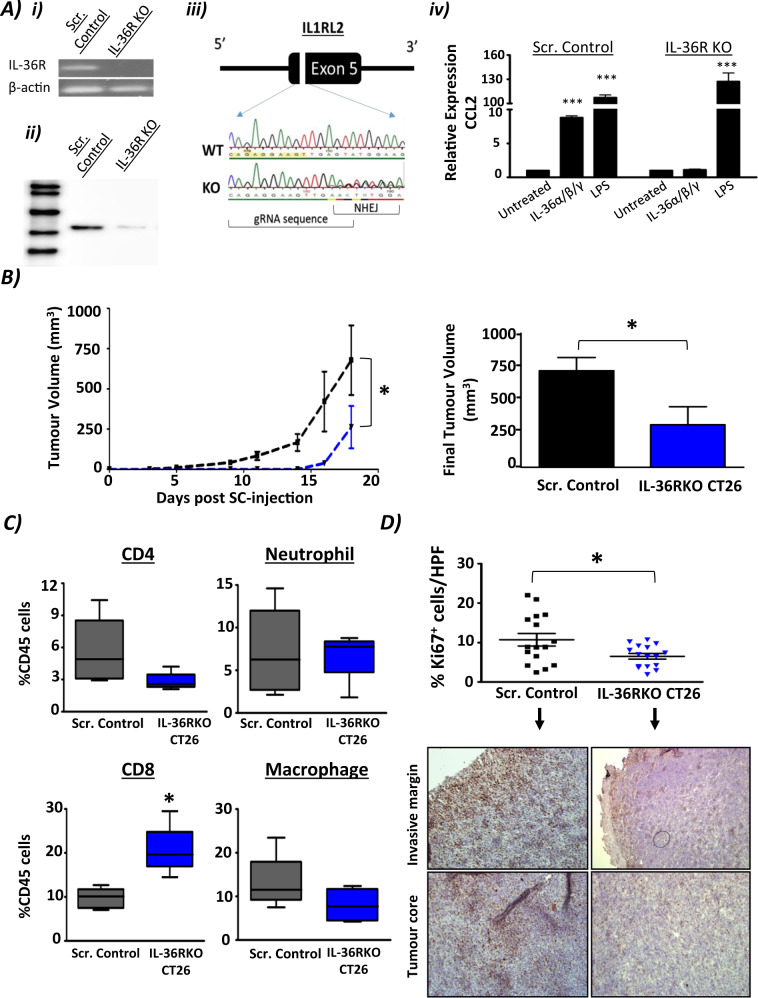
Knockout of IL-36R expression in CT26 cells decreases tumour burden in vivo. **A** IL-36R Knockout CT26 cells were generated by CRISPR-Cas9 technology. This was confirmed by (i) PCR detection of exon 5 of IL-36R, (ii) western blotting for IL-36R protein, (iii) sanger sequencing of exon 5-spanning primer products with TIDE analysis and (iv) functional analysis of cellular response to IL-36 agonist stimulation by CCL2 gene expression in scramble control cells and IL-36R knockout cells. **B** Scramble control or IL-36R KO CT26 cells (2 × 10^5^) were subcutaneously inoculated into BALB/c mice (*n* = 6 per group) and tumour growth measured as previously described. Data points represent the mean value ± SEM with final tumour volumes also shown. **C** Tumour tissue was extracted, processed and analysed for immune cell infiltration by flow cytometric analysis for CD4 T cells (CD4+/CD8−), CD8 (CD4−, CD8+), Neutrophils (CD45+, LY6G+), and Macrophage (CD45+, F4/80+) quantification. **D** Tumour tissue was extracted, processed, paraffin embedded and immunostained with anti-Ki67 and images were taken of invasive margins and tumour cores and quantified by ImageJ software. Representative images are shown (mag = 40X; Scale 100 µm). Statistical analysis was performed by One-way ANOVA with Dunnetts post hoc test (**A**) and student T Test (**B**, **C**, **D**).

In order to further examine the potential mechanisms of action by which IL-36R signalling in colon cancer cells affects tumorigenesis, IL-36R KO CT26 cells were generated using CRISPR/Cas9 technology. Single cells clones were isolated by FACS and knockout of IL-36R was confirmed by both mRNA (Fig. 6Ai) and Western blotting (Fig. 6ii). Sanger sequencing with subsequent TIDE analysis of gRNA target sites revealed complete KO knockout of IL-36R in our clones (Supplementary Fig. S2). Consistent with a lack of IL-36R expression, IL-36RKO cells showed a lack of induction of CCL2 transcription in response to IL-36R agonist stimulation (Fig. 6A iv). Balb/c mice were subcutaneously injected with scramble control CT26 cells or IL-36RKO CT26 cells and tumour growth was monitored. All mice developed palpable tumours. However, tumour growth was significantly decreased in the IL-36RKO group in comparison to the scramble control group (Fig. 6B). Immune infiltrate analysis of the tumours showed a decrease in CD4^+^ T cell and macrophage infiltration in the IL-36RKO tumours in comparison to scramble control tumours. A significant increase in CD8^+^ T cells was also detected in IL-36RKO tumours, with no significant change detected in neutrophil infiltration (Fig. 6C). Tumour tissues were also processed, embedded and immunostained with anti-Ki-67 in order to assess the proliferative state of the tumour cells in both groups. A significant reduction of Ki-67 expression was observed in IL-36RKO tumours in comparison to scramble control tumours at both the invasive margins and in the tumour core (Fig. 6D). These findings suggest that blocking of IL-36R signalling by administration of IL-36Ra and knockout of IL-36R expression in tumours results in different immune infiltrate profiles of tumours; however, both were capable of significantly reducing cellular proliferation of tumour cells.

## Discussion

In this study we have demonstrated the increased expression of IL-36 family members in CRC tumour tissue relative to adjacent non-tumour tissue in our own tissue biobank as well as in a large scale microarray dataset. We have shown that IL-36R^+^ CRC cell lines respond to IL-36 agonists by induction of pro-tumorigenic gene expression, increased cellular proliferation, migration and invasion in vitro. We have utilised the CT26 mouse colorectal tumour model to demonstrate that inhibiting IL-36R signalling by administration of IL-36Ra, or by CRISPR Cas9-mediated IL-36R knockout in tumour cells, results in a reduced tumour burden in mice with a reduction in tumour cell proliferation, indicating that targeting of the IL-36R in CRC may have potential therapeutic benefits.

Several recent studies have demonstrated IL-36 signalling to be an effective method to augment the immune response to improve tumour rejection. This has been achieved by manipulation of the immune system with IL-36R agonists to target immune populations, resulting in immune cell proliferation and activation (9-12, 23, 24). Although this has been shown to be beneficial in several cancer studies, our data shows that there may be important bystander effects associated with IL-36 signalling on tumour cells. Our in vitro investigations showed very high levels of induction of well-characterised pro-tumorigenic genes in CRC cell lines after stimulation with IL-36R agonists. Each of these genes, with the exception of TGFβ (the lowest induction levels observed), have been strongly implicated as pro-tumorigenic chemokines in the context of CRC with known abilities to induce tumour cell migration, proliferation, immunosuppressive leukocyte recruitment, promotion of angiogenesis and chemoresistance [22–28]. Of particular note, CXCL-1 was significantly induced in both human and murine cells, with this chemokine highly implicated in the pathogenesis of CRC, playing a role in the adenoma-adenocarcinoma sequence (77% of cases), angiogenesis and recruitment of pro-tumorigenic stromal cells [29]. This significant induction of pro-tumorigenic genes highlights the necessity to consider signalling by the IL-36R^+^ tumour cells when considering IL-36 adjuvant therapies, as well as consideration of the importance of blocking this signalling pathway to reduce the pro-tumorigenic effects of these chemokines.

Several studies have reported a role for IL-36R signalling in the induction of cellular proliferation across several cell types. This proliferative effect has been observed in CD4^+^ andCD8^+^ T cells and NK cells [30] following IL-36R stimulation, but IL-36R stimulation has also been seen to drive proliferation of T-regulatory cells, cytokine-derived keratinocytes, intestinal epithelial cells and colonic subepithelial myofibroblasts (31-33). Our study shows signalling through the IL-36R also increases cellular proliferation in CRC cell lines, with each agonist showing different potencies in driving this effect in 2D models. Our 3D models reflected these findings, with IL-36R agonists clearly shown to increase cellular proliferation and aid spheroid growth. Therefore, the ability of IL-36 to drive cellular proliferation is now evident across a wide range of cell types and as such, this function of IL-36 must be now considered in all biological contexts. These findings align with previous studies showing IL-36 signalling to not be limited to inflammation and reflect other IL-1 family members in their multi-functional roles in cellular processes [31].

In addition to cellular proliferation, we have identified a key role of IL-36 in driving colon cancer cell migration and invasion. IL-1 family members have long been recognised as pluripotent signalling molecules which may contribute to many of the hallmarks of cancer including metastasis. Studies of IL-1 signalling showed that inhibition of IL-1R signalling by IL-1Ra could reduce liver and lung metastasis in B16 murine melanoma tumours, with similar findings more recently reported in a mouse model of breast cancer [32, 33]. Furthermore, IL-1β treated normal colonic myofibroblasts have been demonstrated to be able to induce CRC cell line migration in vitro, further highlighting the complex nature of IL-1 signalling and cellular migration [34]. Our study shows both IL-36β and IL-36γ are able to potently stimulate cellular migration and invasion in human and murine CRC cells, both of which are hallmarks of metastasis. Further elucidation of migration-associated gene expression patterns are required to understand through which pathways IL-36R signalling mediates this pro-tumorigenic property. It may be of benefit to complete more long-term metastatic CRC models in vivo in order to confirm the clinical relevance of IL-36 signalling in cancer cell migration and invasion.

Previous studies to date concerning IL-36 in tumorigenesis have predominantly focused on the effectiveness of IL-36R agonists in stimulating cells within the TME to favour tumour rejection by immune populations such as CD4^+^, CD8^+^ T cells and NK cell through activation and/or proliferation [35]. Other beneficial mechanisms reported include the formation and maintenance of tertiary lymphoid organ structures within the TME, resulting in increased dendritic cell-mediated tumour antigen presentation and T cell priming [11]. Our in vivo CT26 tumour model reflects these findings, as we have shown that i.p. administration of IL-36R agonist cocktail reduces tumour burden. However, we have also clearly shown that IL-36Ra treatment more significantly reduces tumour burden than administration of the IL-36 agonists. These potentially contradictory findings were accompanied by evidence of divergent mechanisms of tumour reduction. I.p. administration of IL-36 agonists was seen to be associated with a significant increase in CD4^+^ and CD8^+^ T cells within the tumour, whilst no significant alterations of these immune populations were observed in IL-36Ra treated mice. In contrast, immunostaining of tumour tissue of IL-36Ra treated group tumours revealed a significant decrease in Ki-67+ cells, which was not observed in the IL-36 agonist treated groups. Given the clear proliferative role of IL-36R agonists as determined by our in vitro findings, it is reasonable to conclude that IL-36Ra acts to reduce tumour burden by inhibition of IL-36-mediated proliferation of tumour cells. It is of interest to speculate as to why previous studies involving tumour cells engineered to overexpress the IL-36 agonists [9, 12], showed tumour reduction via modification of the TME and did not show any effect on tumour proliferation as may have been anticipated from our findings. Of note, the cells engineered to overexpress IL-36 agonists in those experiments included breast and melanoma cancer cell lines with the expression status of the IL-36R in those cell lines not investigated. In addition, it is possible that the pro-tumorigenic functions for IL-36 signalling described here may be specific to colon cancer cells. Indeed, a recent study investigating the role of IL-36γ supports our findings for a pro-tumorigenic function for IL-36 receptor signalling. Using both the IL-36γ and the IL-36Ra knock-out mice, these authors demonstrate that, in the absence of IL-36γ signalling, the incidence of colon tumorigenesis was reduced in the AOM/DSS, AOM/Vil-Cre;Trp53fl/fl and ApcMin/+ models of colon cancer. In contrast, the incidence of tumorigenesis is increased in the absence of the IL-36Ra [20]. Further work is required to identify whether the pro-tumorigenic effects observed in both this study, and our findings, is tumour type specific and also to determine the relative contribution of IL-36 signalling in both the immune and tumoral compartments.

Whilst each of the IL-1 family members has been shown to have pro and anti-tumorigenic effects to date, IL-36-related studies to date have shown primarily anti-tumorigenic roles [31, 36]. Given the complex divergent nature of IL-1 family members across many pathologies including cancers, it is of little surprise that IL-36R signalling may indeed have both pro- and anti- tumorigenic effects, especially in a complex disease such as CRC. However, as observed here, administration of IL-36Ra reduced tumour burden to a much more significant extent than administration of the IL-36 agonists. Furthermore, this finding was repeated in mice wherein IL-36Ra was administered once tumours had become palpable. This too showed a more significant reduction in tumour burden in IL36Ra-treated groups than in the IL-36 agonist treated group. Not only does this demonstrate that the IL-36Ra may have potential as a component of a therapeutic regimen for tumour reduction, but also that it may have more effective therapeutic benefit than administration of IL-36 agonists. This finding is in keeping with other studies investigating targeting of the IL-1R in tumorigenesis, where blocking of either agonists or receptor members of this family has been shown to result in tumour reduction. In the Canakinumab Anti-inflammatory Thrombosis Outcomes Study (CANTOS), inhibition of the IL1β inflammatory pathway by Canakinumab has been shown to significantly reduce lung cancer incidence and mortality [37] and similarly, administration of IL-1Ra reduced liver and lung metastasis [32, 33]. As a strong overlap in response between IL-36 cytokines and IL-1β has been observed, it is perhaps not surprising that blocking IL-36R signalling may have a similar anti-tumorigenic response to that observed following blocking of the IL-1R [38].

In conclusion, therefore, our findings not only indicate a pro-tumorigenic function for IL-36 signalling in colon cancer cells, and that administration of IL-36Ra merits further investigation for its therapeutic benefit in CRC. Due attention must be paid to the dual functions of IL-36 signalling in tumorigenesis when considering targeted therapy strategies for CRC patients. Such targeted therapies may greatly benefited from patient stratification according to IL-36R tumour expression status, as seen with other therapy strategies [39].

## Methods and methods

### Study populations

The study protocol, including all procedures and study populations has been previously described [40]*.* In brief, the study was approved by the University College Cork Clinical Research Ethics Committee of the Cork Teaching Hospitals (ECM (3) P 3 September 2013). For patient cohort 1, 24 fresh samples of human colon cancer and paired normal tissues were collected in RNALater and stored at −20 until processing. For patient cohort 2, 66 samples were fixed in formalin and embedded in paraffin for subsequent IHC analyses. All samples were obtained during surgery at the Mercy University Hospital Cork following informed consent. Details on patient demographics are contained in Supplementary Table S1.

### Cell line maintenance

Cell lines were obtained from ATCC and maintained in DMEM supplemented with 10% FCS and 1% penicillin/streptomycin solution in a 37 C, 5% CO_2_ humidified incubator unless otherwise stated.

### Mice and tumour model

Female BALB/c mice (4–6 weeks) were obtained from Envigo UK (Oxon, UK) and experiments performed in accordance with ethical approval. Animals were randomly assigned to treatment groups. To establish subcutaneous tumours, mice were injected into the right flank with 1 × 10^5^ CT26 cells suspended in 200 μl phosphate-buffered saline. Tumour growth was monitored by regular measurement of tumour length (a) and width (b) using a Vernier calliper, and the volume was calculated as follows: ½ × (a × b^2^). Animals were culled 28 days later or upon reaching a humane endpoint during monitoring. The acceptable maximum tumour diameter for mice to be culled was 1.5 cm, in accordance with the ethical guidelines. Tumours were excised and processed for subsequent analysis. All treatments were administered in a blinded fashion.

### Generation of IL-36R KO CT26 cells

CRISPR/Cas9-mediated knockout of the IL-36R in the murine colon cancer cell line CT26 was accomplished using IL-1Rrp2 (IL1RL2) CRISPR/Cas9 KO Plasmid (m) and IL-1Rrp2 HDR Plasmid (m) (sc-430727 and sc-430727-HDR; Santa Cruz Biotechnology, Dallas, TX, USA) or a Control CRISPR/Cas9 Plasmid (sc-418922; Santa Cruz Biotechnology) according to the supplier’s protocol. Transfected cells were selected in normal growth medium containing 0.5 mg/mL Puromycin (Thermo Fisher Scientific, Waltham, Massachusetts, United States) for 2 to 3 weeks. Single cell clones were isolated by Aria Fusion Cell sorter, and IL1RL2 knockout clones screened by Western blotting for IL-36R. Cells were also stimulated with IL-36R ligands to confirm loss of function, and gRNA site sequences were Sanger sequenced. Sequencing chromatograms were analysed using the TIDE web tool (https://tide.nki.nl/) to assess the percentage of indel mutant allele vs. WT allele.

### Quantitative real-time PCR

Where indicated in the figure legends, cells were stimulated for 4 h with recombinant cytokines (Supplementary Table S3). Total cellular RNA was isolated using the GenElute Mammalian Total RNA Mini Kit (Sigma–Aldrich, Dorset, UK) according to the manufacturer’s instructions. cDNA was synthesized using the Bioline kit (London, U.K.). RT-PCR was performed using the LightCycler480 System (Roche, West Sussex, U.K.). Individual PCR primer pairs and probes were designed using the Roche Universal Probe Library Assay Design Centre (www.roche-applied-science.com/sis/rtpcr/upl/adc.jsp). Primer sequences and probe combinations are provided in Supplementary Table 2. All samples were run in triplicate, and relative quantitation was calculated using the 2^−ΔΔCt^ method. Transcript levels were normalized to the amount of β-actin mRNA, and expression levels shown as fold induction relative to untreated.

### ELISA

Cells were stimulated for 24 h with recombinant cytokines (Supplementary Table S3), cell culture supernatant was removed, and protein concentration quantified according to the manufacturer’s instructions. In particular, the concentration of CXCL1, CCL2 and CCL20 (R&D Systems, Minneapolis, Minnesota, USA).

### Immunohistochemical analysis

Immunohistochemical staining was performed on formalin-fixed, paraffin-embedded (FFPE) sections as previously described [41] with tissues sections re-hydrated through a series of ethanol washes followed by heat-mediated antigen retrieval and antibody incubation as outlined in Supplementary Table S4. Parallel negative controls were performed for each antibody, using rabbit IgG or mouse IgG instead of primary antibody. IL-36 antibody specificity was further confirmed by pre-adsorbing the primary antibodies for IL-36α/β/γ for 1H with a 10:1 excess (5 μg/ml recombinant protein) of recombinant ligand to antibody (Supplementary Fig. S1). For the IL-36 family member expression in Fig. 1, a semi-quantitative scoring system was used as per previously published (20). In brief, 5 HPF images were taken per specimen with each image given a score of negative, weak, moderate or strong according to the intensity of staining of the normal adjacent epithelial and tumour cells. Sections were scored in a blinded fashion by EB and AH and the average score assigned. The staining intensity for Ki67 expression was determined using ImageJ software (National Institutes of Health, Bethesda, MD; http://imagej.nih.gov/ij/, 1997–2012). Specifically, per tumour, 5 HPF images (40x magnification) were taken both at the invasive margin and tumour core, and the average number of Ki67^+^ cells per tumour was determined.

### BrdU proliferation assay

HT29 cells (2.5 × 10^3^/well) and CT26 cells (5 × 10^3^/well) were seeded in 96 well plates and serum starved(DMEM) for 24 h. Fresh media with test reagents was added, and 24 h later, cell proliferation was measured through BrdU incorporation using the BrdU Cell Proliferation Assay Kit (Cell Signalling Technology, Beverly, MA), according to the manufacturers protocol. Absorbance was measured using the Promega GloMax^®^Multi Detection System. Experiments were repeated three times and each experiment was performed in triplicate.

### Resazurin assay

HT29 cells (2.5 × 10^3^/well) and CT26 cells (5 × 10^3^/well) were seeded in 96 well plates and serum starved for 24 h. Fresh media was then added along with the indicated stimulant/control. 24 h later, cells were washed, media supplemented with 44 µM resazurin was added, and resazurin reduction to resorufin measured fluorometrically using a GENios plate reader (TECAN, Grodig, Austria) and Xfluor spreadsheet software. Results obtained were normalised relative to the untreated cells. Experiments were repeated three times and each experiment was performed in triplicate.

### Flow-cytometry

Tumours were extracted from mice and tumour tissue was enzymatically digested with Collagenase II (1 mg/mL) and DNAse I (100 Kunitz/mL) for one hour at 37 °C. Cells were then passed through a 70 µm cell strainer and suspended in RBC lysis buffer (Biolegend, San Diego, California, U.S) for 10 minutes on ice. 1 × 10 ^ 6 cells were added to each FACS tube and blocked with anti-mouse CD16/32 (Biolegend) for 10 min on ice. Cells were then stained with a cocktail of antibodies or appropriate isotypes (Supplementary Table S4) and LIVE/DEAD Green fixable Dead cell stain (L34970, ThermoFisher). Sample data collection was performed using BD LSRII Flow Cytometer (BD Biosciences, Franklin Lakes, NJ, USA) and data was analysed using FlowJo™ Software (Becton, Dickinson and Company; 2019). The gating strategy is shown in Supplementary Fig. S3.

### Migration assays

Cell migration was determined by wound scratch assay using IBIDI culture inserts (IBIDI GmbH). 70 µl of a 4 × 10^5^ cells/ml solution were added into the two reservoirs of the same insert and incubated at 37 °C/5% CO_2_ overnight. The insert was gently removed creating a gap of ∼500 µm and images of cell migration were taken using BRESSER MikrOkular Full HD eyepiece until the 500 µM gap had been filled. Media was changed every 72 h with fresh stimulant added appropriately. The in vitro migration assays were also conducted utilizing Transwell inserts (Falcon^®^; BD Biosciences). Experiments were completed as outlined by Pijuan et al. using chambers of Matrigel coated membranes (coated with 5 mg/ml Matrigel Matrix [BD Biosciences] for cell invasion assay) or uncoated membranes (for cell migration assay) [42]. Cells in 5 different fields were counted under a microscope at ×40 magnification connected to a digital camera with which images were captured. The experiments were repeated three times.

### 3D spheroid generation

3D spheroid generation was performed using CT26 cells (2.5 × 10^3^/mL) or HT29 cells (5 × 10^3^/mL). 200 µL of these suspensions were added to agar-coated wells of round-bottom 96 well plates and subsequently centrifuged for 20 min at 300 g and incubated in a humidified incubator at 37 °C. Cells were allowed to aggregate for 72 h and only wells containing one single spheroid were then proceeded with for experimentation. 100 µL of media was then removed and replaced with 100 µL of fresh media with or without stimulant every 72 h. Images were obtained using an inverted microscope with a digital camera. Images were quantified using the SpheroidJ plugin in the ImageJ software suite (National Institutes of Health, Bethesda, MD; http://imagej.nih.gov/ij/, 1997–2012).

### Western blotting

Cells were lysed for 1 h on ice in ice-cold lysis buffer containing 50 mM Tris-HCl (pH 8.0), 150 mM NaCl, and 1% Triton X-100, supplemented with complete protease inhibitors (Roche Diagnostics). The protein content of each sample was analysed using the BCA Protein Assay Kit (Pierce, Rockford, IL). Equal amounts of protein were separated on a 10% SDS–polyacrylamide gel and transferred to nitrocellulose membranes. Membranes were probed overnight at 4 °C with primary Antibody (See Supplementary Table S4). As an internal control, all membranes were subsequently stripped of the first Ab and re-probed with anti–β-actin–specific Ab (Sigma–Aldrich). Protein bands were analysed using ImageJ (National Institutes of Health, Bethesda, MD; http://imagej.nih.gov/ij/, 1997–2012). Changes in protein expression were determined after normalizing the band intensity of each lane to that of β-actin.

### Public transcriptomic data sets

Transcriptomic data from colorectal cancer tumors was downloaded from Gene Expression Omnibus (accession code GSE68468). Affymetrix microarray data from the Gene Expression Omnibus was analysed for IL-36R expression in normal colon mucosa, polyp tissue and colon cancer tissue. The data from Affymetrix Human Genome U133 Plus 2.0 Array was processed by MAS5.0 post-processing.

### Statistical analysis

Results were statistically evaluated using one-way ANOVA with a Tukey post-test, or by the Student paired t test, unless otherwise stated. The *p* < 0.001 are indicated by three asterisks (***). The *p* < 0.01 are indicated by two asterisks (**). The *p* < 0.05 are indicated by one asterisk (*).

## Supplementary information

Supplemental Table 1

Supplemental Table 2

Supplemental Table 3

Supplemental Table 4

Supplemental Figure 1

Supplemental Figure 2

Supplemental Figure 3

Supplemental Figure legends
