# Supplementary material for: IL-36 signalling enhances a pro-tumorigenic phenotype in colon cancer cells with cancer cell growth restricted by administration of the IL-36R antagonist
Source: Oncogene. 2022 Apr 1;41(19):2672–84. doi: 10.1038/s41388-022-02281-2 (PMC9076531; doi:10.1038/s41388-022-02281-2)
Supplement: Supplementary file 2 — Supplemental Table 2 [file 41388_2022_2281_MOESM2_ESM.docx]

Table S2. List of qRT-PCR primers/ primer sequences

| **Human** |  |  |
| --- | --- | --- |
| **Gene** | **Forward (5’-3’)** | **Forward (5’-3’)** |
| IL36β | TGAAGACATCATGAACCCACA | TGTCGAGAATCACGAATAGCA |
| IL36γ | AAGTGACAGTGTGACCCCAGT | GGATTCTGGATTCCCAAATAAA |
| IL36R | GCGTGTCAAGCCATACTGAC | CCTCCATATCCAGCTCTTTCTG |
| IL36RN | GAGGAACAGGCAGACTCCAC | R CAATGCCGAGTCCTTCATTC |
| CXCL1 | TCCTGCATCCCCCATAGTTA | CTTCAGGAACAGCCACCAGT |
| CCL20 | TTGCTCCTGGCTGCTTTGAT | AGTCAAAGTTGCTTGCTGCT |
| CXCL8 | GAGCACTCCATAAGGCAC | ATGGTTCCTTCCGGTGGT |
| TNFα | CGCTCCCCAAGAAGACAG | AGAGGCTGAGGAACAAGCAC |
| Β-actin | ATTGGCAATGAGCGGTTC | GGATGCCACAGGACTCCA |
|  |  |  |
| **Murine** |  |  |
| **Gene** | **Forward (5’-3’)** | **Forward (5’-3’)** |
| CXCL1 | AGCCTCTAACCAGTTCCAGC | CTGGGATCATGGTGCTGTGT |
| CCL2 | TCACTGAAGCCAGCTCTCTCT | GTGGGGCGTTAACTG |
| CCL5 | AATCCCCTACTCCCACTCGG | TCTTGGGTTTGCTGTGCAGA |
| TGFβ | CTCCGCTGACTCTCTTGG | AGGTGGTCGCAAAAACGA |
|  |  |  |
| **Other primers** |  |  |
| **Gene** | **Description** |  |
| hIL-36α | 137628 custom Taqman assay |  |
| mIL-36α | mm00457645_m1 |  |
| mIL-36β | mm01337546_m1 |  |
| mIL-36γ | mm00463327_m1 |  |
| mIL-36R | Mm00519245_m1 |  |
| mIL-36RN | Mm01333586_m1 |  |
