# Supplementary material for: IL-36 signalling enhances a pro-tumorigenic phenotype in colon cancer cells with cancer cell growth restricted by administration of the IL-36R antagonist
Source: Oncogene. 2022 Apr 1;41(19):2672–84. doi: 10.1038/s41388-022-02281-2 (PMC9076531; doi:10.1038/s41388-022-02281-2)
Supplement: Supplementary file 3 — Supplemental Table 3 [file 41388_2022_2281_MOESM3_ESM.docx]

Table S3. List of recombinant cytokines.

| **Recombinant protein** | **Supplier** | **Cat. no** |
| --- | --- | --- |
| Recombinant Mouse IL-36α (carrier-free) | Biolegend | 555904 |
| Recombinant Mouse IL-36β (carrier-free) | Biolegend | 554504 |
| Recombinant Mouse IL-36γ (carrier-free) | Biolegend | 552804 |
| Recombinant Mouse IL-36Ra (IL-1F5) (carrier-free) | Biolegend | 760804 |
| Recombinant Human IL-36α (carrier-free) | Biolegend | 551602 |
| Recombinant Human IL-36β (carrier-free) | Biolegend | 761104 |
| Recombinant Human IL-36γ (Animal-Free) | Biolegend | 711602 |
| Recombinant Human IL-36Ra (IL-1F5) (carrier-free) | Biolegend | 760904 |
