# Supplementary material for: IL-36 signalling enhances a pro-tumorigenic phenotype in colon cancer cells with cancer cell growth restricted by administration of the IL-36R antagonist
Source: Oncogene. 2022 Apr 1;41(19):2672–84. doi: 10.1038/s41388-022-02281-2 (PMC9076531; doi:10.1038/s41388-022-02281-2)
Supplement: Supplementary file 4 — Supplemental Table 4 [file 41388_2022_2281_MOESM4_ESM.docx]

Table S4. List of antibodies

| **Target Protein** | **Supplier** | **Cat no.** |
| --- | --- | --- |
| **Immunohistochemistry** |  |  |
| IL-36α | Abcam | ab180909 |
| IL-36β | Abcam | ab180890 |
| IL-36γ | Lifespan Biosciences | LS-C338023 |
| IL-36R | Abcam | ab180894 |
| Ki67 | Abcam | ab16667 |
| Secondary biotinylated anti-Rabbit | Dako | E0432 |
| Secondary biotinylated anti-Rat | Vector | BA-9400 |
|  |  |  |
| **Western blotting** |  |  |
| IL-36R | Invitrogen | PA587629 |
| Phospho-p42/44 human | Cell Signalling | 9102s |
| Total p42/44 human | Cell Signalling | 9102 |
| Phospho-AKT human | Cell Signalling | s473 |
| Total AKT human | Cell Signalling | 9272s |
| Phospho-PI3K p85α | Invitrogen | PA5-105116 |
| Total PI3K p85α | R&D Systems | MAB2998 |
|  |  |  |
| **Flow Cytometry** |  |  |
| LIVE/DEAD™ Fixable Green Dead Cell Stain Kit, for 488 nm excitation | Invitrogen | L34970 |
| LIVE/DEAD™ Fixable Near-IR Dead Cell Stain Kit, for 633 or 635 nm excitation | Invitrogen | L10119 |
| Alexa Fluor® 700 anti-mouse CD45 | Biolegend | 103128 |
| APC anti-mouse CD3 | Biolegend | 100236 |
| Brilliant Violet 605TM anti-mouse CD4 | Biolegend | 100548 |
| APC/Cyanine7 anti-mouse CD8a | Biolegend | 100714 |
| PE anti-mouse Ly-6G | Biolegend | 127608 |
| Brilliant Violet 421TM anti-mouse F4/80 | Biolegend | 123132 |
