# Supplementary material for: IL-36 signalling enhances a pro-tumorigenic phenotype in colon cancer cells with cancer cell growth restricted by administration of the IL-36R antagonist
Source: Oncogene. 2022 Apr 1;41(19):2672–84. doi: 10.1038/s41388-022-02281-2 (PMC9076531; doi:10.1038/s41388-022-02281-2)
Supplement: Supplementary file 5 — Supplemental Figure 1 [file 41388_2022_2281_MOESM5_ESM.pptx]

## Slide 1
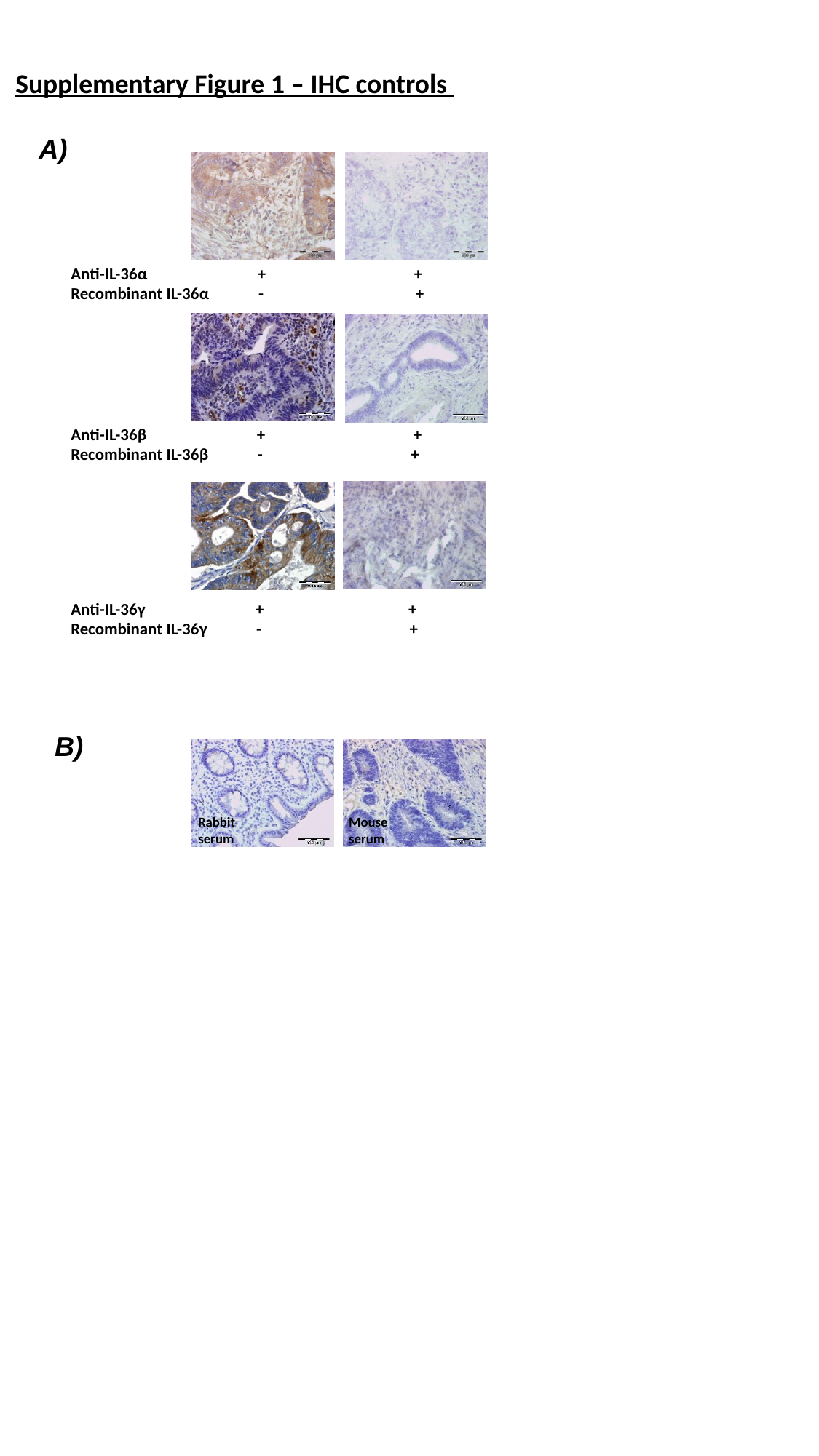

Supplementary Figure 1 – IHC controls
A)
Anti-IL-36α + +
Recombinant IL-36α - +
Anti-IL-36β + +
Recombinant IL-36β - +
Anti-IL-36γ + +
Recombinant IL-36γ - +
B)
Rabbit serum
Mouse serum
