# Supplementary material for: IL-36 signalling enhances a pro-tumorigenic phenotype in colon cancer cells with cancer cell growth restricted by administration of the IL-36R antagonist
Source: Oncogene. 2022 Apr 1;41(19):2672–84. doi: 10.1038/s41388-022-02281-2 (PMC9076531; doi:10.1038/s41388-022-02281-2)
Supplement: Supplementary file 6 — Supplemental Figure 2 [file 41388_2022_2281_MOESM6_ESM.pptx]

## Slide 1
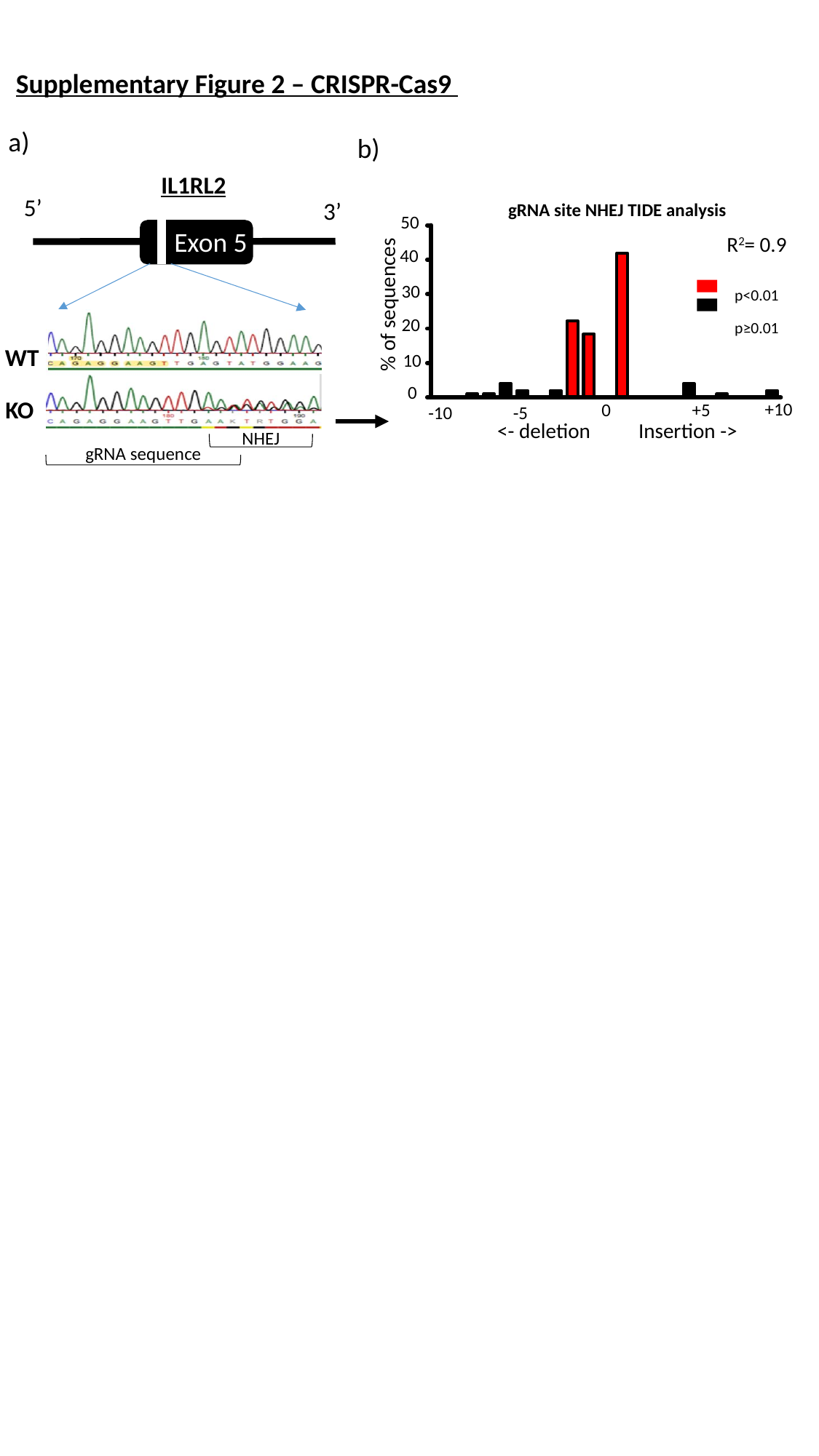

Supplementary Figure 2 – CRISPR-Cas9
a)
b)
IL1RL2
5’
3’
gRNA site NHEJ TIDE analysis
50
Exon 5
R2= 0.9
p<0.01
p≥0.01
40
30
% of sequences
20
WT
10
0
KO
+10
0
+5
-10
-5
<- deletion
Insertion ->
NHEJ
gRNA sequence
