# Supplementary material for: IL-36 signalling enhances a pro-tumorigenic phenotype in colon cancer cells with cancer cell growth restricted by administration of the IL-36R antagonist
Source: Oncogene. 2022 Apr 1;41(19):2672–84. doi: 10.1038/s41388-022-02281-2 (PMC9076531; doi:10.1038/s41388-022-02281-2)
Supplement: Supplementary file 7 — Supplemental Figure 3 [file 41388_2022_2281_MOESM7_ESM.pptx]

## Slide 1
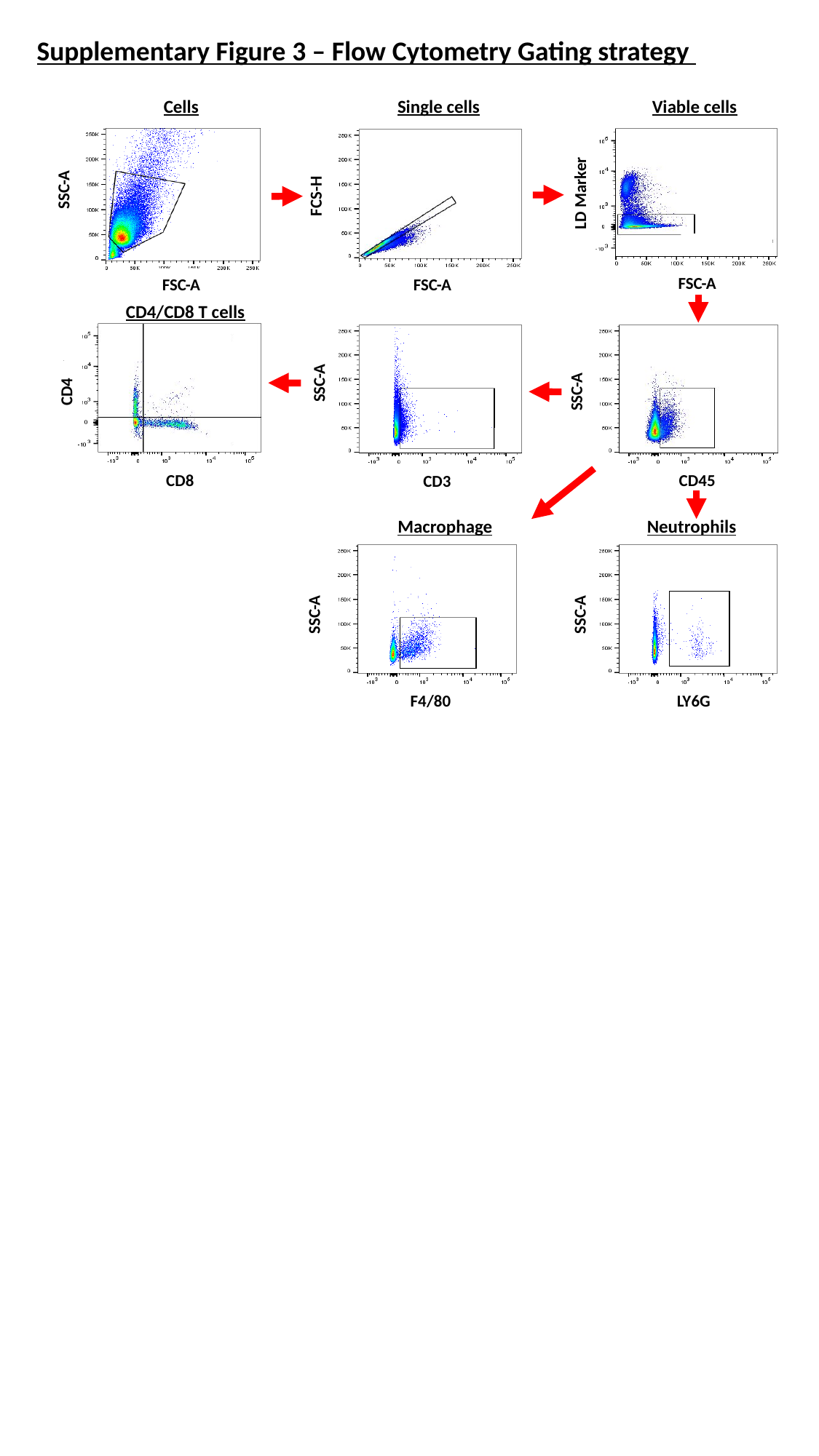

Supplementary Figure 3 – Flow Cytometry Gating strategy
Cells
Single cells
Viable cells
SSC-A
LD Marker
FCS-H
FSC-A
FSC-A
FSC-A
CD4/CD8 T cells
SSC-A
CD4
SSC-A
CD45
CD8
CD3
Macrophage
Neutrophils
SSC-A
SSC-A
F4/80
LY6G
