# Supplementary material for: IL-36 signalling enhances a pro-tumorigenic phenotype in colon cancer cells with cancer cell growth restricted by administration of the IL-36R antagonist
Source: Oncogene. 2022 Apr 1;41(19):2672–84. doi: 10.1038/s41388-022-02281-2 (PMC9076531; doi:10.1038/s41388-022-02281-2)
Supplement: Supplementary file 8 — Supplemental Figure legends [file 41388_2022_2281_MOESM8_ESM.docx]

**Supplemental Figure Legends:**

**Figure S1:** Immunohistochemical Controls. (A) The recombinant proteins for the respective IL-36 agonists were included at 5μg ml−1 during primary antibody incubation in control staining for the specificity of the antibodies. (B) Parallel negative controls were performed for each antibody, using rabbit (IL-36α, IL-36β) IgG or mouse (IL-36γ) IgG instead of primary antibody. Representative micrographs are shown. Original magnification: × 400.

**Figure S2.** CRISPR-Cas9 TIDE analysis. Sanger sequences of gRNA target sites of clones were analyzed by TIDE analysis to confirm no residual WT sequence was remaining in clones.

**Figure S3.** Flow cytometry gating strategy. Dissociated mouse tumor tissues were blocked and incubated with a master mix of antibodies as described in Table S3. The gating strategy above was applied to detect CD4+ T cells, CD8+ T cells, Macrophage (CD45+, F4/80+) and Neutrophils (CD45+, LY6G+).
